# Immunometabolism In Brain Aging and Neurodegeneration: Bridging Metabolic Pathways and Immune Responses

**DOI:** 10.14336/AD.2024.1293

**Published:** 2025-12-18

**Authors:** Shokofeh Rahimpour, Briana L. Clary, Sanaz Nasoohi, Yohanna S. Berhanu, Candice M. Brown

**Affiliations:** ^1^Department of Microbiology, Immunology, and Cell Biology, School of Medicine, West Virginia University, Morgantown, WV 26506, USA.; ^2^Department of Neuroscience, School of Medicine, West Virginia University, Morgantown, WV 26506, USA.; ^3^Rockefeller Neuroscience Institute, West Virginia University, Morgantown, WV 26506 USA

**Keywords:** metabolism, microglia, astrocytes, Alzheimer’s disease, Parkinson’s disease, multiple sclerosis

## Abstract

The complex set of interactions between the immune system and metabolism, known as immunometabolism, has emerged as a critical regulator of disease outcomes in the central nervous system. Numerous studies have linked metabolic disturbances to impaired immune responses in brain aging, neurodegenerative disorders, and brain injury. In this review, we will discuss how disruptions in brain immunometabolism balance contribute to the pathophysiology of brain dysfunction. The first part of the review summarizes the contributions of critical immune cell populations such as microglia, astrocytes, and infiltrating immune cells in mediating inflammation and metabolism in CNS disorders. The remainder of the review addresses the impact of metabolic changes on immune cell activation and disease progression in brain aging, Alzheimer’s disease, Parkinson’s disease, multiple sclerosis, stroke, spinal cord injury, and traumatic brain injury. Furthermore, we also address the therapeutic potential of targeting immunometabolic pathways to reduce neuroinflammation and slow disease progression. By focusing on the interactions among brain immune cells and the metabolic mechanisms they recruit in disease, we present a comprehensive overview of brain immunometabolism in human health and disease.

## 1. Introduction

In recent years, immunometabolism has become an increasingly significant field of research, revealing complex relationships between the metabolic mechanisms that regulate cellular pro-inflammatory and anti-inflammatory responses. In order to maintain specific cellular functions, metabolic reprogramming is shaped to support individual cellular metabolic needs. Chronic neuroinflammation and metabolic dysfunction are the hallmarks of neurodegenerative diseases [1]. The central nervous system’s (CNS) metabolic niche is distinguishable from others in the periphery based on its unique nutrient environment, selectivity of the blood-brain barrier, and interaction between infiltrating innate and adaptive leukocytes [2]. Unfavorable environments induce two types of maintenance programs: homeostasis and stress. Both processes are part of maintenance but use different metabolic pathways. Energy conservation, catabolic metabolism, and resistance to environmental stress are all features of homeostasis programs. Protective responses against pathogens and other hostile environmental factors are powered by anabolic metabolism, whereas stress programs consume a large amount of energy. For instance, immunity to pathogens depends on anabolic processes related to glucose and glutamine utilization. Leukocyte proliferation and biosynthesis of defense-related proteins require these programs. By applying this framework to the overall immune response, it becomes evident why metabolic reprogramming at both the cellular and organismal levels is crucial to the host's response to an infection. Cellular uptake and utilization of readily available glucose is integral in fulfilling the high energy demands of immune cells needed to mount a robust immune response. Biomolecules such as cytokines, antimicrobial proteins, and lipid mediators are synthesized during immune cell activation.

**Figure 1.**
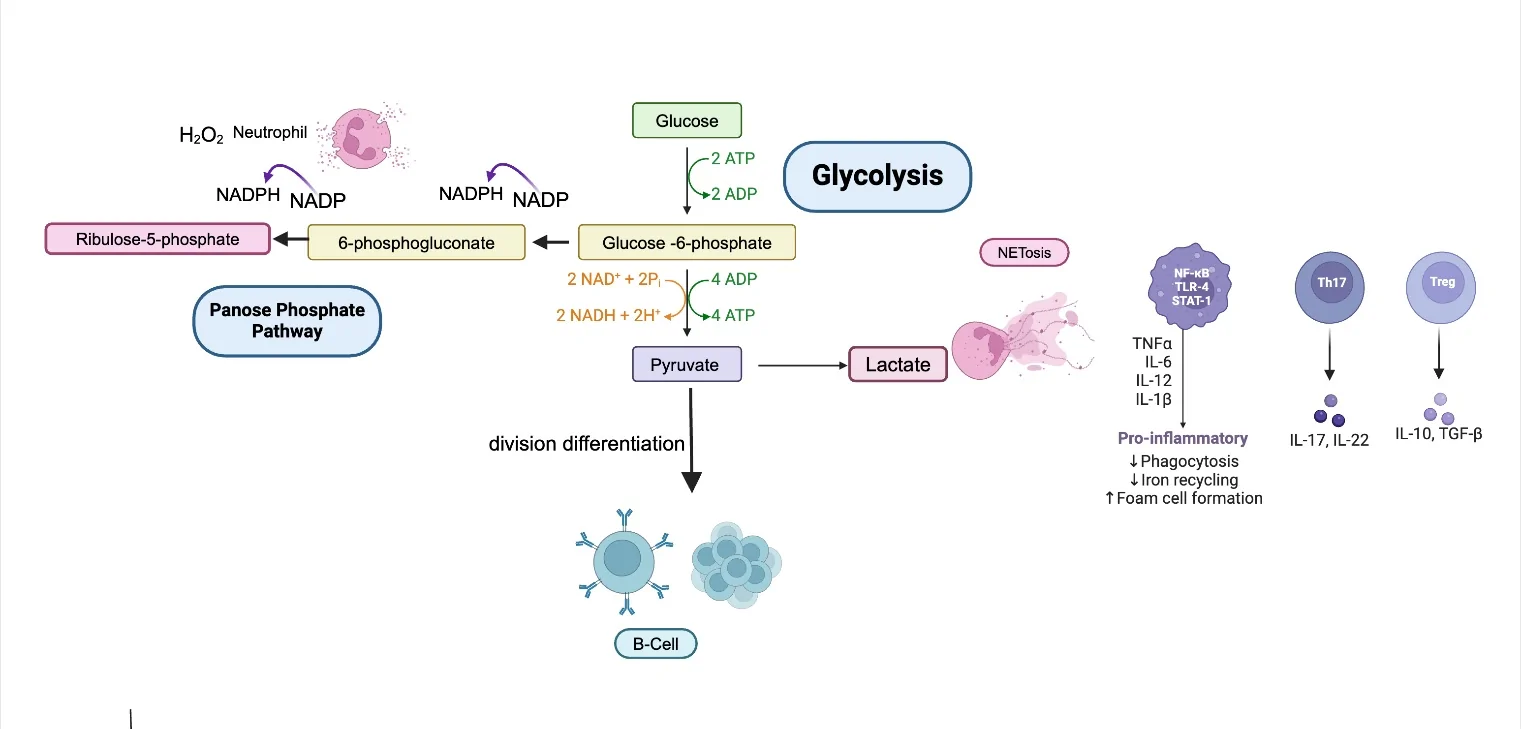
**Overview of glycolysis and pentose phosphate and immune cell signaling pathways in brain immunometabolism**. The diagram depicts various glycolytic pathways and processes that are related to cellular metabolism and immunological responses. Glycolysis involves taking glucose from the environment to produce pyruvate and other biosynthetic intermediates. Lactate production from the glycolysis pathway causes NETosis, a form of cell death involving neutrophils, and can also activate macrophage, Th17, and T regulatory pathways. Pyruvate, a glycolysis intermediate, is involved in B cell differentiation and division. The primary role of the pentose phosphate pathway is to support cell proliferation and survival in the cytosol. The pentose phosphate pathway transforms intermediates from glycolysis into nucleotides and amino acids. By producing NADPH, the pathway maintains H_2_O_2_ production by neutrophils. Abbreviations in figure: NET: neutrophil extracellular traps; NAD: nicotinamide adenine dinucleotide; TNFa: tumor necrosis factor-α; NF-κB: nuclear factor-κB; TLR4: toll-like receptor 4; STAT1: signal transducer and activator of transcription 1; TGFβ: transforming growth factor β.

**Figure 2.**
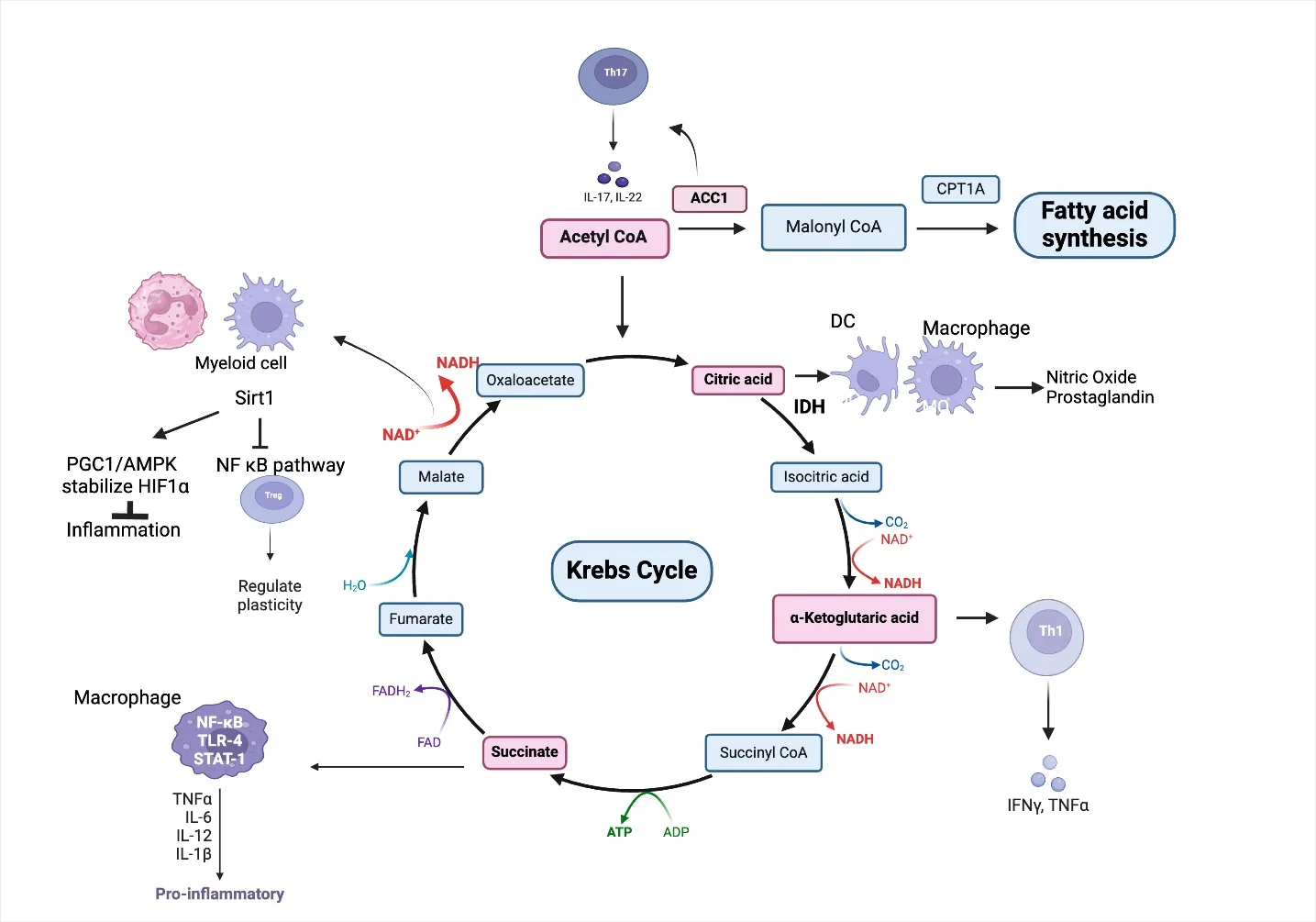
**Overview of oxidative phosphorylation, fatty acid synthesis, and immune cell signaling pathways in brain immunometabolism**. Oxidative phosphorylation (OXPHOS) is also known as the TCA cycle or Krebs cycle. In the TCA cycle, glucose-derived pyruvate and fatty acids are converted into acetyl coenzyme A (acetyl-CoA). The TCA cycle generates ATP by transferring electrons from NADH and FADH2. Acetyl CoA is the initiator of the Krebs cycle. By converting citric acid into isocitric acid, isocitrate dehydrogenase (IDH) induces macrophages and dendritic cells to release nitric oxide (NO) and prostaglandins. Ketoglutarate stimulates TH1 cells to produce interferon-γ (IFNγ) and tumor necrosis factor-α (TNFα.) Succinates influence important signaling pathways that initiate inflammatory responses in immune cells, specifically causing macrophage activation. NADH produced in TCA cycle is a cofactor in redox reactions and a co-substrate for sirtuins (Sirt1) and ribosyl transferases. Sirt1 suppresses inflammation and regulates the plasticity of helper T regs. Acetyl-CoA and acetyl-CoA carboxylase 1 (ACC1) in the fatty acid synthesis pathway promote Th17 cell development by producing phospholipids for cellular membranes.

Similarly to the immune system, glucose is an essential nutrient needed for appropriate brain function. Neural functions consume around 25% of the body's glucose and 20% of its oxygen, while the brain only accounts for about 2% of the total weight of the human body [3]. As a result, the brain is highly sensitive to changes in energy supply, with even minor changes in energy processing linked to impaired brain function. As summarized in Figure 1, the most efficient way to utilize glucose energy is through glycolysis, which generates pyruvate in the tricarboxylic acid (TCA or Krebs) cycle [1, 4]. Likewise, nicotinamide adenine dinucleotide (NADH) and flavin adenine dinucleotide (FADH2) are redox cofactors produced in the TCA cycle and serve as proton donors for mitochondrial electron transport and respiration/oxidative phosphorylation (OXPHOS), which is summarized in Figure 2. In total, 36 adenosine triphosphates (ATPs) are generated per glucose molecule through a series of enzymatically-regulated steps.[5] Several pathways are needed to maintain a healthy immune system, including glycolysis, OXPHOS, fatty acid oxidation (FAO), and the pentose phosphate pathways. Immune cells undergo metabolic reprogramming in response to stress or activation to provide the energy for their effector functions. In many leukocytes, anaerobic glycolysis (i.e., Warburg metabolism) is strongly stimulated for rapid ATP generation and nucleotide synthesis. On the other hand, the anti-inflammatory responses tend to polarize toward OXPHOS.

## 2. Overview of immunometabolism

Despite their appearance as binary switches, metabolic pathways are dynamic systems that exhibit extensive flux between the different types of pathways and stimuli. During disease progression and severity, peripheral leukocytes exhibit oxidative stress, decreased activity of lysosomal proteins, and an imbalance in lipid metabolism. The same changes in these pathways are also observed in the brain. For example, following brain injury, peripheral leukocytes with altered metabolic profiles might be more pathogenic and interact differently with resident glia and neurons. However, it remains unclear whether these immunometabolic changes are responsible for the disease or are merely indications. Understanding metabolic biases is crucial for regulating cell function and modifying the progression of neurodegenerative diseases. This review focuses on the mechanisms of underlying immunometabolic changes that are associated with neurodegenerative disorders. The transition in immune cells from a homeostatic state at baseline to an active state during inflammation and back to a memory state illustrates the importance of metabolism in fueling these cellular processes. These transitions are heavily influenced and regulated by cellular sensors, metabolic intermediates, and metabolic regulators. Some of these critical factors are described in the following sections.

### 2.1 Primary metabolites and immune cell sensors

In both active innate and adaptive immune responses, immune cells utilize metabolic components differently based on their functional objectives. The primary metabolites involved in brain immunometabolism are summarized in Table 1 and will be referenced throughout the review. ATP is necessary for maintaining the basic functions of all cells, whether they are quiescent, replicating, or activated. The glycolytic, TCA cycle, and OXPHOS pathways are interconnected to supply these bioenergetic requirements [6]. During homeostasis, and particularly during inflammation, there is a high variability in available oxygen and nutrients which, in turn, increases the demand for metabolically active immune cells. The immune system has evolved a variety of metabolic programs to accommodate both changing and challenging metabolic conditions. These metabolic programs provide the cells with cellular energy and biomolecules they need to overcome these challenges [7].

Most of the inflammatory cells found in inflammatory lesions are recruited rather than being resident immune cells. The shifts seen in tissue metabolism result from the profound recruitment of inflammatory cell types, cell interaction, and proliferation of innate and adaptive immune cells. An innate immune response involves the recruitment of neutrophils and monocytes to the site of inflammation [8, 9]. In contrast, an adaptive immune response involves the proliferation of T cells and B-cells and has significantly different metabolic demands. In this context, it is essential to understand the interactions between metabolic triggers in the microenvironment, such as glucose, oxygen, and ATP, and molecular mechanisms that promote immune cell recruitment and activation [7]. Toll-like receptors (TLRs) are the most widely distributed type of pattern recognition receptors (PRRs) in immune cells, and they initiate the innate immune response by regulating cell metabolism. TLR signaling activates dendritic cells (DCs) in response to bacterial toxins and other environmental danger signals, including cellular damage. Lipopolysaccharides (LPS) binding to TLRs have been associated with changes in metabolic programming from mitochondrial OXPHOS, fueled by fatty acid oxidation, to aerobic glycolysis, resulting in DC activation[10]. These pathways are illustrated in Figure 2.

**Table 1:** Key Metabolites and Signaling Molecules Implicated in Brain Immunometabolism.

Metabolite	Metabolic Pathway	Immune cell sources	Effectors	Function(s)	References
Pyruvate	Glycolysis		N/A	Oxidized through the tricarboxylic acid cycle	[147]
			N/A	Substrate for numerous metabolites	
Lactate	Glycolysis	Macrophages	HIF-1α activation	Suppresses inflammatory response	[147]
		Neutrophils	N/A	Activates NETosis	
		Macrophages	NF-κB inhibition	Inhibits Inflammasome activation inflammasome pathway in macrophages.	
		Treg T-cells	IL-17 activation	Reduces migration and migration promotes Treg differentiation.	
		T-cells		Retains autoreactive T cells at the site of inflammation	
Succinate		Macrophages	HIF-1α activation	Upregulation of IL-1β	[148]
Itaconate	TCA cycle	Macrophage	Nrf2	Antioxidant and anti-inflammatory activity	[149]
			N/A	Inhibition of succinate dehydrogenase (SDH)	
Citrate	TCA cycle	Activated macrophages and dendritic cells	N/A	Substrate for NO and prostaglandins	[148]
			Acetyl-CoA	Prevents activation of IFNg	
Nicotinamide adenine dinucleotide (NAD+)		Myeloid cells	N/A	DNA repair, epigenetic regulation	[150, 151]
			sirtuins and ribosyl transferases	Co-factor in redox reactions and a co-substrate for sirtuins and ribosyl transferases	
			Sirt1	Suppresses the NF κB pathway in myeloid cells and promotes metabolic reprogramming through PGC1*/AMPK and HIF1α stabilization to prevent inflammation.	
			Sirt1	Regulates the plasticity of helper T cells and Tregs	
α-ketoglutarate (αKG)	Citric acid cycle	CD4+ T cells	N/A	Promotes CD4(+) T cell differentiation	[152]
Acetyl coenzyme A (acetyl-CoA) carboxylase 1 (ACC1)	Fatty acid synthesis	Th-17 T cells	N/A	Promotes Th17 cell development	[153]
NADPH oxidase	TCA cycle	Neutrophils	N/A	Metabolizes oxygen into neutrophil microbicidal product H_2_O_2_	[154]

There is a wide distribution of cytokines secreted by immune cells and cytokine receptors on immune cells. Cytokines are signaling polypeptides that modulate biological and metabolic processes through cell surface receptors. In response to infections or inflammation, pro- and anti-inflammatory cytokines are produced by increasing NF-κB expression to modify metabolism activities. For instance, a simulation of the Interleukin-2 and Interleukin-2 receptor (IL-2-I/L2R) signal promotes glucose metabolism, which, in turn, promotes T cell differentiation. Interterlukin-4 and Interleukin-4 receptor (IL-4-I/L4R) increase glucose transporter-4 (GLUT4) expression, enhancing lipid and glucose metabolism. A combination of IL-10 and IL-10R enhances insulin sensitivity, inhibits aerobic glycolysis, and stimulates OXPHOS. Signaling from interferons and interferon regulatory factors (IFNs-IRFs) stimulates FAO and reduces lipid biosynthesis [11].

Additionally, lymphocyte activation through T and B cell receptors (TCRs/BCRs) is associated with major metabolic challenges in neurodegenerative diseases such as multiple sclerosis (MS) and Alzheimer's disease (AD). TCR signaling leads to metabolic reprogramming of naive T cells. In response to TCR ligation, glucose, and amino acid transporters coordinate to facilitate nutrient transport and T cell development. Transcriptional factor c-Myc is activated by TCR through an increase in glycolysis metabolism gene expression. Moreover, catabolic pathways of ATP generation, such as fatty acid β-oxidation, are actively suppressed by c-Myc expression[12]. Alternatively, the BCR complex plays a central role in antigen-specific clonal expansion and directs antibody production against newly encountered antigens. BCR signaling and collaboration with cell surface receptors results in several adaptations, such as increasing glucose uptake and glycolysis. This glycolytic pathway is highlighted in Figure 1. This is in part due to the increased amount of GLUT1 transporters, in addition to the increased downstream demand for glucose-6-phosphate. Furthermore, stimulation of IL-4, BCR cross-linking, and LPS and TLR4 engagement on B cells have been shown to induce glucose oxidation along with increased pyruvate synthesis [13].

### 2.2 Signaling pathways employed by immune cells to respond to metabolic shifts

There are several conserved signaling pathways implicated in immunometabolism. Activation of neutrophils via pathogen-associated molecular patterns (PAMPs) and chemokines triggers glycolysis, glutaminolysis, reactive oxidative species (ROS) production, and inflammation. Through PAMP-TLR recognition, dendritic cells become activated, thereby leading to an increase in glycolysis and cytokine production and presentation. Inflammatory macrophages of the innate immune system distinguish non-self from self through utilizing pattern recognition receptors (PRRs) (i.e., TLRs and nucleotide-binding oligomerization domain (NOD) proteins) to identify PAMPs. Likewise, hypoxia-inducible factor 1-alpha (HIF-1α) regulates macrophage immune responses through increasing glycolysis and glutaminolysis, resulting in ROS and cytokine production and enhanced M1 polarization. Furthermore, anti-inflammatory macrophages can activate peroxisome proliferator-activated receptors (PPARs) and PGC1α to cause differentiation and increase survival in these cells. Lastly, TCR signaling pathways, through CD3 and CD28, activate c-Myc, leading to glycolysis, glutaminolysis, and mitochondrial OXPHOS. This activation results in increased proliferation and cytokine secretion. Memory CD8+ T cells utilize IL-15, IL-15R, and tumor necrosis factor receptor-associated factor 6 (TRAF6), which promote survival. When B cells are activated by antigen, PAMP-BCR, PRRs (TLRs) increase glycolysis and induce antibody secretion and proliferation [6].

In this section we have provided an overview of the major immune cells and metabolites that are involved in brain immunometabolism, and we have discussed the receptors and signaling pathways that generate immunometabolic responses. The remainder of the review will focus on immunometabolic mechanisms in brain aging, neurodegenerative disorders, and brain injury.

## 3. Brain aging

Our bodies are prone to chronic low-grade inflammation as we age, which can lead to metabolic diseases and neurodegenerative disorders [14]. Various cellular stresses or macromolecular damage can induce irreversible cell cycle arrest, resulting in cellular senescence, preventing proliferation into damaged tissue, and oncogenesis [15]. Normally, immune cells remove these senescent cells. Consequently, senescence-associated secretory products (SASPs), such as IL-1β, IL-6, monocyte chemoattractant protein-1 (MCP-1), and high mobility group box 1 (HMGB1), are produced in large amounts when the cell is unable to proliferate [16-18]. In addition, SASPs have been implicated in the hypersecretion of growth factors, proteases, and other signals that promote chronic inflammation, immune-surveillance, and fibrotic formations [19, 20]. The increased expression of inhibitory molecules, alterations in cell major histocompatibility complex (MHC), and reduced cell cytotoxicity seen in aging can dysregulate immunosurveillance and clearance, leading to the accumulation of senescent cells [18].

### 3.1 Metabolic changes in brain aging

A decline in nicotinamide adenine dinucleotide (NAD) is a common mechanism that is often observed during the aging process. SASPs activate tissue-resident cells and infiltrating immune cells, and they also regulate the metabolism of NAD, a key energy metabolite [14, 21, 22]. Different metabolic changes in NAD can modulate cellular senescence. CD38, a NAD-ase present on the surface of cells, including immune cells, is stimulated by SASP factors and is responsible for aging-related declines in NAD levels [23]. In the short term, lower NAD/NADH levels contribute to mitochondrial dysfunction, sustained activation of the AMP-activated protein kinase (AMPK) signaling pathway, and, ultimately, send more cells into senescence. The AMPK pathway is thought to play a protective role by maintaining energetic stasis, modulating inflammatory marker release, reducing the production of ROS, enhancing autophagy, and slowing the aging process. Additionally, p53 activation during the AMPK pathway prevents IL-1-dependent SASP release of IL-10, CCL27, and tumor necrosis factor-α (TNF-α), thus promoting adipocyte and keratinocyte differentiation. With chronic senescent cell accumulation, there is an increase in NAD/NADH levels, but less activation of the AMPK pathway and more genotoxic stress. This lack of AMPK activation allows NF-kB to have an unimpeded pro-inflammatory response and increased cell survival, resulting in increased inflammatory conditions [24, 25].

**Figure 3.**
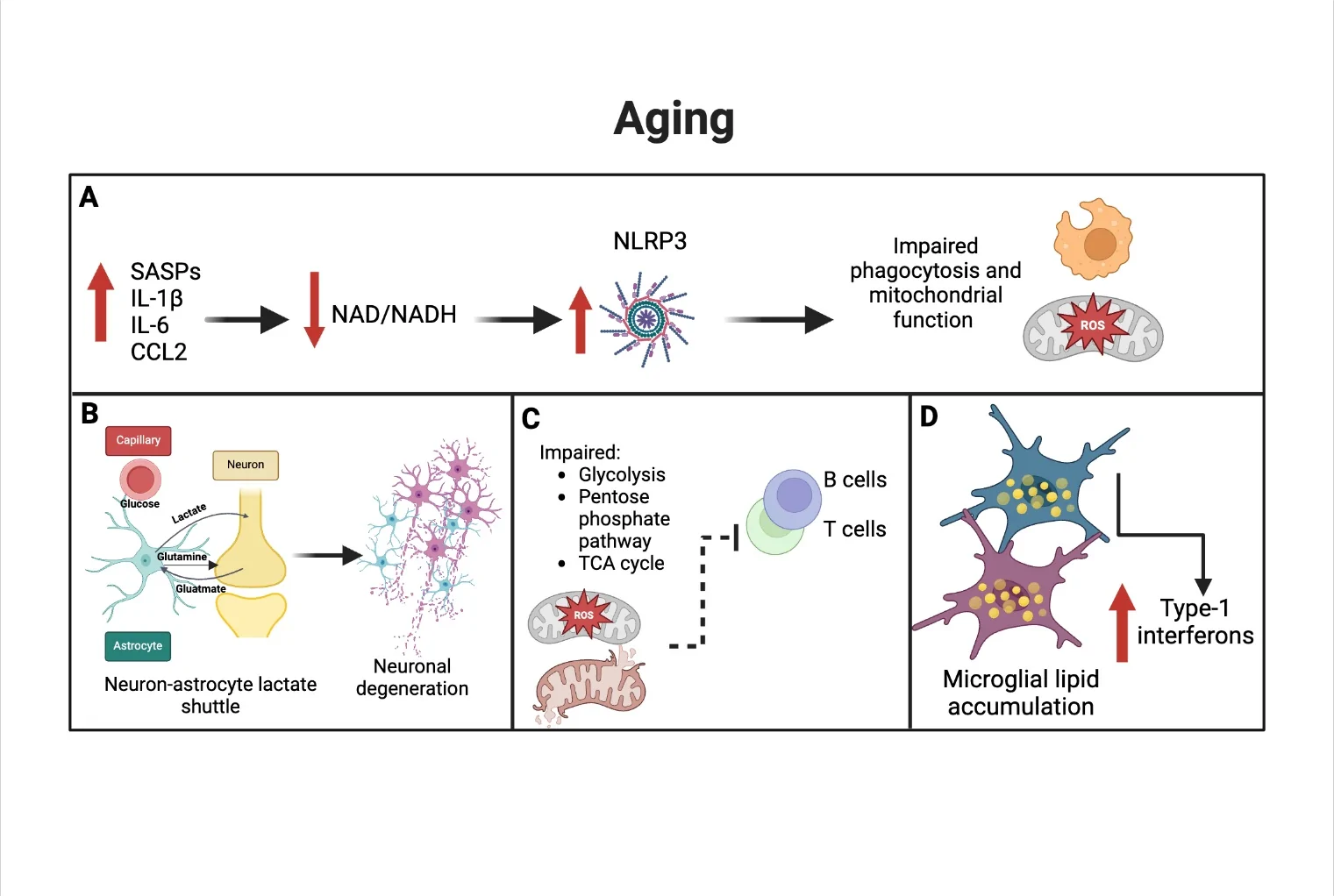
**Interplay of cellular metabolism and immune activation in aging and neurological disorders**. The diagram depicts the metabolic and immunological alterations linked to aging conditions. Each segment is interconnected, highlighting the significance of metabolic dysregulation and immune responses. (**A**) Senescence-associated secretory phenotypes (SASPs) exhibit increased levels of interleukin-1β, IL-6, and monocyte chemoattractant protein-1 (MCP-1). SASPs also regulate NAD/NADH levels. Lower NAD levels in aging activate the inflammasome in macrophages, leading to mitochondrial dysfunction and an inflammatory phenotype. (**B**): A decrease in astrocyte numbers and dysfunction weakens the lactate shuttle, leading to neuronal degeneration and accelerated aging. (**C**) Aging reduces glycolytic activity and mitochondrial energy production, leading to the buildup of reactive oxygen species (ROS). These changes impair B cell function, hinder plasma cell differentiation, and affect tissue homing and memory T cell differentiation. (**D**) Aged microglia show significant lipid droplet accumulation under steady-state conditions, which is linked to exacerbated type I interferon responses.

As humans age, the mitochondrial genome accumulates more genetic mutations, leading to mitochondrial heterogeneity. Mitochondrial heterogeneity has been identified as a critical factor contributing to microglia heterogeneity, differences in aging outcomes, and changes in medication response [26]. Within the innate immune system the NLRP3 inflammasome acts as a sensor for any potential threats. Inflammasome activities are also known to contribute to long-term inflammation associated with aging, both in the body and in the brain, as well as the potential driving force of insulin resistance, cardiovascular disease, and neurodegenerative diseases, such as AD. Normally, these sensors become activated in response to the release of damage-associated molecular patterns (DAMPs). Subsequently, NOD-like receptor protein 3 (NLRP3)-dependent caspase-1 is activated, leading to the secretion of major inflammatory cytokines, IL-1b and IL-18. This secretion results in a host of downstream effects potentiating the sterile inflammation and tissue remodeling seen during the aging process. Likewise, DAMPs tend to accumulate in the aged brain, therefore reigniting this pathway repeatedly. In an older brain, resident macrophages and inflammatory pathways, including activation of the NLRP3 inflammasome, are elevated; however, phagocytosis, antigen presentation, metabolism, and mitochondrial function are impaired. A notable decrease in NAD production can be seen in aged macrophages, which increases inflammatory response and dysregulates oxidative metabolism [27-29]. The collective mechanisms that link diminished NAD production with activation of the NLRP3 inflammasome are summarized in Figure 3A.

The dysregulation of astrocyte-mediated glycolysis is another mechanism commonly observed in aging and neurodegenerative diseases. Astrocytes aid in the regulation of neuronal metabolism and neurovascular coupling in order to provide energy for neuronal activity.[30] As shown in Figure 3B, the uptake of glucose and release of lactate are triggered by synaptic glutamate absorption by astrocytes by the neuron-astrocyte lactate shuttle [31]. In terms of regulating glycolysis and inhibiting the accumulation of oxidative stress, neurons primarily rely on mitochondrial OXPHOS. Because of this, they are more at risk of mitochondrial dysfunction related to aging. In contrast, astrocyte dysfunction causes neurons to degenerate, resulting in both normal aging and neurodegeneration. A decrease in the number of astrocytes and the weakening of the lactate shuttle are also important causes of aging [32-34]. Overall, these studies suggest that astrocyte dysfunction contributes to pathological aging of the nervous system and, as a result, may accelerate the aging process.

### 3.2 Immune changes in brain aging

In addition to secreting antibodies, B cells also produce plasma cells that regulate other immune cells through the release of anti- and pro-inflammatory cytokines. Chronic, low-grade inflammation associated with brain aging, is often amplified by the plasma cells' production of IL-1, IL-6, and TNF-α. The release of these inflammatory cytokines favors myelopoiesis and inhibits lymphopoiesis, thus reducing the number of B cells.[35, 36] Recent discoveries have also connected TLR as one of the key players in age-related impairment, due to its overexpression in older plasma cells [37-39]. Other key mediators secreted by aged B cells include IL-4 and IL-10, which contribute to the decline in lymphopoiesis, diminished immune response to antigen challenge, and nonresponsive B cell receptors post-TLR activation [37, 40]. In addition, metabolic changes in B cells play an important role in the decline of immunity in aging. Typically, activated B cells consume more glucose and amino acids, perform more glycolysis, and have a higher activity of pyruvate dehydrogenase, which works to improve the efficiency of the TCA cycle and supports the high energy demands of B cell division and differentiation with acetyl-CoA derivatives [35]. B cells also exhibit prolonged non-energy-stress-related activation of AMPK, which allows for the accumulation of synthesized lipids that are not normally seen in other immune cells [34].

Conversely, in aging, there is a slight decrease in glycolytic activity, mitochondrial energy production, and increased accumulation of mitochondrial ROS. As shown in Figure 3C, these metabolic events disrupt B cell function, impede their differentiation into plasma cells, and promote immunoglobulin class switching [41]. Aged antibody-secreting cells have also been shown to have decreased fatty acid uptake, along with less expression of metabolism genes, including Sirtuin 1 (SIRT1), Forkhead Box O1 Protein (FOXO1), and carnitine palmitoyl transferase 1 (CPT-1), which causes a decrease in mitochondrial activities. This further confirms that the diminished cell response to stimulation, impaired function with energy-intensive processes, and decreased specific antibody production are associated with aging [41, 42].

As we age, we are more likely to develop autoimmunity and infection due to our reduced thymic output, the oligoclonality of our T cell repertoire, an overall reduction in the number of naïve T cells, and the accumulation of highly differentiated T cell subsets with impaired pro-inflammatory functions [43]. Amongst the minority of aged naïve T cells remaining, aside from metabolic-induced impaired activation, there is a disruption to the one-carbon metabolism, which distinctly limits differentiation and survival [44]. Enhanced mammalian target of rapamycin (mTOR) signaling via miR-21 in aged T cells also affects tissue homing and memory cell differentiation.[45, 46] A decrease in T cell-dependent antibodies responsive to vaccination is associated with an increase in IL-10-producing T follicular helper cells with age [47]. Interestingly, alpha-ketoglutarate, a metabolite of the TCA cycle, induces aged T cell production of IL-10 [48].

Lastly, changes in microglial lipid accumulation have also been shown to contribute to brain aging, as many studies have shown that a precise regulation of lipid accumulation is critical for maintaining brain homeostasis, as described in Figure 3D. The accumulation of lipid droplets can enhance pro-inflammatory responses in microglia [49]. Aged microglia show significant lipid droplet accumulation under steady-state conditions, which is linked to exacerbated type I interferon responses and altered immunometabolism following ischemia. Microglial depletion and repopulation using colony-stimulating factor 1 receptor (CSF1R) antagonists in aged mice decreased lipid droplet accumulation, attenuated innate immune responses, and improved motor function after stroke. Transcriptomic analyses also revealed that repopulated microglia exhibited reduced pro-inflammatory signaling and enhanced metabolic function [50].

In conclusion, aging is associated with significant changes in the metabolism of immune cells in the brain. These changes can result in alterations in the functioning of the immune system, leading to an increased susceptibility to infections, chronic inflammation, and neurodegeneration. While the exact mechanisms underlying these changes are still not fully understood, research suggests that a combination of genetic and environmental factors, including diet, lifestyle, and exposure to certain pathogens, can play a role in modulating the metabolism of immune cells in the brain during aging. As such, it is important to continue to investigate and identify potential interventions to help prevent or mitigate the effects of age-related changes in brain immune cell metabolism and associated neurological disorders.

## 4. Multiple sclerosis

Multiple sclerosis (MS) is characterized by demyelination and inflammation of the central nervous system [51]. In MS, activated immune cells attack the brain, causing a wide range of damage to myelin basic protein (MBP) as well as neuronal loss in the central nervous system. Microglia activation, blood-brain barrier dysfunction, and altered endothelial cell activity are crucial features of MS [52]. There are multiple types of immune cells found in the MS brain, but autoreactive T cells are thought to be the primary mediator of inflammation, with some involvement from autoreactive B cells. These peripherally activated lymphocytes enter the CNS, resulting in major destruction to the white matter of the brain through their production of pro-inflammatory cytokines, including IL-1, IL-6, IL-12, IL-17, IL-22, anti-myelin basic protein antibodies, interferon-γ (INF-γ) TNF-α, and chemokine attraction of macrophages via C-C chemokine receptor 2 (CCR2). The interactions of these immune products with microglia and macrophages facilitate the triggering receptor expressed on myeloid cells 2 (TREM2)-induced clearance of myelin basic protein and the increased production of ROS and reactive nitrative species (RNS), which, in large, keep this damaging inflammatory cycle going. This excessive response eventually causes the destruction of grey matter and irreversible neuronal loss, seen with more long-term MS progression [51, 53]. Many factors regarding MS development and isolated causative gene mutations are still largely unknown; however, there is growing evidence that metabolic changes play a role in MS development.

### 4.1 T cell metabolism in MS

As one of the major drivers of metabolic change, obesity has been proposed as a risk factor for MS. Body metabolism could influence the onset and progression of MS by dysregulation of certain hormones, including leptin, insulin, and ghrelin [54]. Several cytokines and hormones, secreted by tissue-resident cells and neighboring immune cells in response to local antigens, have been identified as possible causes of metabolic reprogramming toward a more short-term fuel supply (i.e., glycolysis) [55, 56]. This impaired energy output leads to a diminished production of T regulatory cells (T regs) and a downregulation in the expression of important receptors like CTLA-4, PD-1, and CD71, which help identify autoimmunity and enhance apoptosis of defective immune cells [57]. Moreover, it’s important to understand that not all cells are created equally. T regs specifically use extracellular fatty acids for energy, whereas CD8+ cells can derive fatty acids from either an extracellular source of glucose or scavenge fatty acids from lysosomes to fuel their energy expenditure. However, T regs do have some metabolic flexibility since they are also able to use glycolysis to support their rapid proliferation, although they will preferentially switch to OXPHOS to maximize their suppressive activity. Other immune cells that also exhibit very specific metabolic needs and are heavily involved in the pathogenesis of MS include Th1 and Th17 cells. Th1 and Th17 cells promote inflammation in the CNS through their interactions with B cells, follicular T helper cells, and resident microglia, thus producing highly inflammatory cytokines and ROS/RNS. In general, glycolysis supports the production of Th17 cells, Th1 cells, and INF-γ. Th1 cells can also utilize glutaminolysis for energy consumption; however, Th17 cells are a bit more convoluted, since they can have either a pro-inflammatory or anti-inflammatory response. In terms of pro-inflammatory cytokine production, Th17 cells will predominantly use OXPHOS. Furthermore, Th17 cells extract fatty acids from their *de novo* synthesis pathway to aid in the production of the inflammatory cytokine IL-17. It is believed that intermediates from fatty acid synthesis bind to the transcription factor RORγt promoter on DNA in these cells, inducing the expression of IL-17. Recent findings in mice studies have unveiled that the RORγt promoter may play a more prominent role in the connection to obesity and Th17-related pathologies [58].

The metabolic reprogramming of T cells is crucial for their proliferation, differentiation, and function [58]. Even in the presence of polarizing cytokines, inhibiting glucose metabolism can inhibit Th1 and Th17 differentiation. The survival and function of T regs are primarily dependent on FAO-driven OXPHOS, so lipid species may provide crucial signals for the survival and homeostatic functions of T reg cells [59]. In MS, as T regs migrate into inflamed tissue to perform their immunomodulatory functions, metabolic pathways that control glycolysis can impact T reg migration and/or self-recognition [60, 61]. With this in mind, 2-deoxyglucose (2-DG) has been found to block glycolysis in animal models of experimental autoimmune encephalomyelitis (EAE) and improve clinical outcomes. Essentially, 2-DG is a glucose molecule analog that differs via its hydrogen substitution at the second carbon. Interestingly, this analog competitively inhibits hexokinase to block the production of glucose-6-phosphate from glucose during the first step of glycolysis [62]. Likewise, it has been shown that mice that overexpress GLUT1 in their T cells have a direct link between dysregulated glucose metabolism and autoimmunity [63]. In vitro, stimulation of T cells with glucose increases IFN-γ and IL-2 production, whereas, deletion of GLUT1 reduces CD4+ T cell glycolysis and differentiation [64]. Researchers have made the connection that, in this context, shifting the focus away from glycolytic activity can help to diminish the proliferation and pro-inflammatory actions of Th1 and Th17 cells, while further enhancing the activity of T regs, thus improving the disease [65].

Besides glucose metabolism, T cell lipid metabolism may play a role in MS pathogenesis [66-68]. The membrane of activated T cells contains more cholesterol and fatty acids, suggesting that altered lipid-mediated signaling may contribute to MS development [69-71]. As discussed previously, Th17 has a unique role in requiring *de novo* synthesis of fatty acids for cellular needs, whereas for T regs this is not required. The buildup of free fatty acids synthesized is highly regulated by the enzyme, acetyl-CoA carboxylase (ACC), which makes it a target of interest with new and upcoming pharmacologic treatments. ACC is an enzyme that converts acetyl-CoA to malonyl-CoA for fatty acid synthesis and has two isoenzyme forms, called ACC1 and ACC2. ACC1 can be more readily found in a free form in the cytosol of lipogenic tissues such as adipocytes and the liver. Conversely, ACC2 is a mitochondrial membrane-bound variation found more often in highly exertive tissues like the heart and muscles. Both isoforms can also exist in T cells. A deficiency in ACC1 has been shown to protect mice from EAE. The absence of ACC1 helped to reduce INF-γ, Th17 cells, and IL-17 synthesis and did not negatively affect T regs even when the cells were polarized toward the Th17 cell type. Moreover, this blockade of *de novo* fatty acid synthesis allows for increases in extracellular fatty acid uptake and an increase in FOXP3+ T regs [72].

### 4.2 Oxidative phosphorylation in MS

During the disease process of MS, shifts in the metabolism, causing an imbalance of energy production and conservation, ultimately cause mitochondrial stress, inflammation, and continual demyelination of axons. Overall, in MS there is an underutilization of OXPHOS and impaired pyruvate metabolism. Given the damage to myelinated cells of the CNS, the body tries to compensate for the energetic deficit caused by an overactive immune system by increasing the amount and metabolic frequency of the mitochondria present in active and inactive lesions. This ultimately worsens the oxidative stress and burnout of the mitochondria. Comparatively, mitochondrial DNA is more vulnerable to damage during times of oxidative stress, such as ROS production, than nucleic DNA is, since it lacks a protective nuclear envelope shielding it from damaging cytosolic enzymes. The inflammation, paired with the over-compensation of the metabolism, causes quick burnout, which could cause irreversible damage to mitochondrial DNA, destruction of important proteins and lipids, and obstruction in cell processes [73]. In MS, there are a variety of brain regions that show downregulation in several subunits of complexes I, III, IV, and V involved in OXPHOS, which worsens the ATP deficit. To maintain the energetic output needed, more glycolytic fluctuations occur, leading to increases in serum lactate and α-ketoglutarate levels, as well as enhanced enolase activity, pyruvate kinase activity, and aldolase activity, both before and after meals containing glucose. On the other hand, some studies believe overconsumption of ATP is seen via the buildup of metabolites from its catabolism in the cerebral spinal fluid (CSF) and the blood of MS patients. Interestingly, anti- glyceraldehyde 3-phosphate dehydrogenase (GAPDH) autoantibodies are present in the CSF, resulting in glycolytic pathway downregulation and neuronal cell death. This impairment of GAPDH function opens up a larger discussion of how MS and other neurodegenerative pathologies like AD, Parkinson’s disease (PD), and Huntington’s disease are more similar than was previously thought [53, 74].

As summarized in Table 2, the current literature demonstrates that the etiology of MS is driven by immunometabolic changes that cause impaired glucose and lipid metabolism primarily in T cells, which, in turn, leads to neurodegeneration. Future studies should address how metabolic perturbations generate and sustain pathogenic T cells, with an emphasis on nutrition depletion or metabolite availability. Incorporating this new knowledge will be a vital step in understanding and implementing improved targeted therapies for MS patients.

**Table 2:** T cell Mediated Immunometabolism in Multiple Sclerosis.

Metabolic change	Outcomes	References
Glycolysis	Diminished production of T regs	[57]
Glycolysis	Increased T reg migration and/or self-recognition	[60]
GLUT1 overexpression	Dysregulated glucose metabolism and autoimmunity in T cells	[63, 64]10/16/2025 1:51:00 PM
Fatty acid synthesis reduction by ACC1 deficiency	Reduced INF-γ levels, IL-17 synthesis, and Th17 cells	[72]

## 5. Alzheimer’s disease

Amyloid-beta (Aβ) plaque deposition and hyperphosphorylated tau neurofibrillary tangles in the brain are key neuropathological hallmarks of Alzheimer's disease (AD) [75]. As previously discussed, ApoE is a lipid transport protein implicated in AD. Genome-wide association studies have indicated that metabolic dysfunction, related to APOE4, is closely linked to chronic neuroinflammation. Dose-dependent inheritance of the APOE4 allele is the strongest genetic risk factor for the development of AD [76]. In addition to a role in brain aging and amyloid plaques, APOE4-associated immunometabolism disturbances may promote synaptic loss and exacerbate the pathological features of aging [77, 78]. Regional studies have revealed that the hippocampus and cortex are particularly susceptible to APOE4's impact on immunometabolism through metabolic reprogramming [76].

### 5.1 Microglia and Macrophages

Alterations in microglial metabolism are thought to contribute to chronic inflammation leading to AD by amplifying inflammatory responses in the brain [79]. As innate resident immune cells, microglia in the brain are responsible for phagocytosis and removing toxic Aβ and tau aggregates that contribute to neurodegeneration. These innate immune cells have both oxidative and glycolytic metabolism genes, and there is usually a switch between these metabolic programs in response to inflammation [80-82]. Microglia preferentially use glucose for energy and express several glucose transporters [83, 84]. Activated microglia rely on glycolysis for ATP production, while non-activated microglia rely on oxidative phosphorylation [85]. During AD, toxic aggregates of Aβ and tau accumulate, which activate microglia into a pro-inflammatory state, leading to alterations in genomic expression. This causes a metabolic shift from OXPHOS to glycolysis, which increases ROS production, mitochondrial fission, and dysfunction [49, 86].

Chronic exposure and insufficient clearance of Aβ and tau lead to an impaired immune response and mitochondrial dysfunction in microglia. This dysfunction is particularly problematic because this cell type typically exhibits very low mitochondrial turnover [87, 88]. This concept has been supported by several recent studies. One study has shown suggests that combining recent anti-amyloid monoclonal antibody therapies with agents targeting dysregulated immunometabolic pathways, such as insulin signaling modulators, could enhance clinical outcomes, improve immune function, reduce inflammation, and slow the progression of both amyloid and tau pathology [89]. Another recent study explained the importance of sex-specific treatment in AD using multi-omics data from human brain samples, and they identified key metabolites and gene networks that differed between male and female microglia [90]. Females with AD showed elevated immune signaling, such as IL-10 and IL-17, and alterations in glutamate metabolism, thereby contributing to decreased microglia-neuron communication [89].

Recent research has linked impaired microglial function with TREM2 in neurodegenerative diseases [77, 91]. TREM2 is a receptor that exists on all myeloid cell types in the periphery and mainly on microglia in the CNS. When microglia are exposed to stress, TREM2 plays a vital role in maintaining their metabolic fitness, enhancing phagocytosis, and inducing Aβ clearance. The prevalence of AD and early-onset dementia is higher in individuals with TREM2 mutations [92-94]. One study examined the effects of the TREM2 T66M mutation on knock-in mice and found an age-dependent decline in microglial activity and a significant drop in cerebral blood flow and glucose metabolism. This suggests that microglia may closely regulate glucose utilization and metabolism in the brain [77, 91].

A more recent study on microglia with TREM2 loss of function (LOF) resulted in survival defects, arrest in a low metabolic state, and accumulated lipids [95, 96]. Some studies aimed to determine whether metabolic deficits caused by TREM2 were a secondary consequence of survival deficits or whether TREM2 specifically activated cellular metabolism. Adding monovalent transferrin receptors (TfRs) to antibody transport vehicles (ATVs) facilitates their transport across the blood-brain barrier (BBB) [97]. Specific metabolic pathways have been discovered to boost energetic capacity through ATV: TREM2. It has been shown that ATV: TREM2 increases microglial respiration by promoting mitochondrial FAO. The ATV: TREM2 system also stimulated glucose oxidation, which demonstrated its flexibility. Observing low FDG-PET signals in both AD patients and TREM2 LOF mice, they hypothesized that *in vitro* activity might correlate with clinical efficacy [91, 98].

With mitochondrial dysfunction, GAPDH is another key mediator that shows both direct and indirect participation in AD pathology. GAPDH is a major enzyme used in glycolysis that reversibly phosphorylates glyceraldehyde phosphate dehydrogenase (G3P) into 1,3-biphosphoglycerate and is a housekeeping gene heavily involved in cell longevity and apoptosis. Amidst all the inflammation and production of ROS, the GAPDH enzyme becomes denatured. When GAPDH becomes denatured, it causes decreased glycolytic activity, increased apoptosis of neurons, and even has direct interactions with Aβ and tau, leading to their aggregation. [53]There are several studies that indicate ROS accumulation and NLRP3 inflammasome activation lead to increased glycolytic activity in the brain, contributing to the pathology associated with AD [99-101]. Macrophage metabolism is influenced by NLRP3 inflammasome activation. Moreover, NLRP3 inflammasomes lead to several downstream effects that cause microglia activation, therefore leading to a switch of their metabolism to glycolysis, which can negatively affect energy-demanding clearing processes, such as phagocytosis [102]. Likewise, a similar inflammatory process can be seen in ischemic stroke in which the hypoxic conditions activate microglia and induce a hyper-glycolytic state that is believed to cause the post-stroke brain damage seen, through lactate accumulation and oxidative stress [103]. Lastly, more recent studies have shown that mitochondrial succinate dehydrogenase (SDH) inhibition reduces the pro-inflammatory metabolic rewiring, thereby limiting cytokine production and oxidative stress in microglia [104].

Ketones have been associated with a protective role in AD by reducing oxidative stress and improving synaptic plasticity [105-108]. During glucose shortages, microglia can utilize ketone bodies as a source of energy. The three main sources of ketone bodies produced by the liver include acetoacetate (AcAc), 3-β-hydroxybutyrate (BHB), and, in the lowest quantity, acetone. Clinically, BHB appears to be the most important ketone concerning neurodegenerative diseases [109]. BHB has been brought to light as one of the key derivatives and modulators of fatty acid metabolism and is thought to have a neuroprotective role in the prevention of various neurodegenerative disorders, including AD. The effects of BHB are facilitated by a G_i_ protein-couple receptor called GPR109A. This receptor is found in both macrophages and microglia. Once bound, BHB causes inhibition of microglial activation, attenuates NF-kB signaling, and decreases the production of proinflammatory enzymes (Cox-2 and iNOS), and cytokines (IL-6, TNF-a, and IL-1b) [110-112]. Additionally, BHB, inhibits NLRP3 inflammasome activation in macrophages and primary microglia, resulting in an anti-inflammatory phenotype. Overall, this process helps to stop the impending damage of inflammation and helps protect the brain cells from damage.

### 5.2 Astrocytes

In contrast to the extensive studies establishing a strong link between microglia and Aβ, fewer studies have examined the effect of astrocytes on Aβ in AD. Two studies have identified a disruption in astrocyte branched chain amino acid metabolism in AD transgenic mice. Neurotransmitters and energy metabolism may be imbalanced due to impaired metabolism of branched amino acids. One study showed that altered glucose metabolism in APPswe/PSEN1dE9 mice resulted in impaired glutamine oxidation and mitochondrial function Another study showed that the hippocampal neurons in 5XFAD mice developed reduced GABA synthesis due to astrocyte TCA cycle and glutamine synthesis impairment [113, 114]. Other ways that astrocytes contribute to immumometabolic responses in AD brain is through dysfunctional cholesterol metabolism. This process promotes the release of oxysterols, cytokines, and chemokines, which results in neuronal damage, and potentially, neuronal cell death [30, 67]. Another way that astrocytes may contribute to AD pathology is through the production of mitochondrial complex III-derived reactive oxygen species (CIII-ROS). Production of CIII-ROS was shown to amplify immunometabolic pathways in astrocytes following stimulation by IL-1α and oligomeric amyloid-beta (oAβ). As a result, CIII-ROS production selectively modified proteins involved in metabolism and immune signaling via NF-κB and STAT3 activation. The study found that reducing CIII-ROS increased lifespan and decreased both neuroinflammation and tau pathology in mouse models of tauopathy [115].

As summarized in Table 3, a growing body of research in both AD transgenic mouse models and in AD patients supports a critical role for immunometabolism in AD pathophysiology. Current studies implicate microglia and macrophages, and to a lesser extent, astrocytes, as the major players. These findings suggest that targeting microglial metabolism, particularly, could help mitigate neuroinflammation in the early stages of AD. Thus, it is plausible to propose that reprogramming microglia to enhance oxidative metabolism may reduce the neuroinflammation seen in AD.

**Table 3:** Cell-specific shifts in immunometabolism during Alzheimer’s disease.

Immune cell	Metabolic change	Species	Experimental Model	References
Microglia	Shift from oxidative phosphorylation to glycolysis	Mouse	APP/PS1 mice 5xFAD mice	[49, 155]
		AD patients		
Microglia	Succinate dehydrogenase (SDH) with dimethyl malonate (DMM)	Mouse	Primary microglia culture from cortical tissue of neonatal mice	[104]
Microglia and myeloid cells	Lipid accumulation and mitochondrial respiration	Mouse (C57BL/6J)	Primary microglia culture	[77, 91, 92, 94]
		Human	HMC3 microglial cells	
		AD mouse models	APP_Swe_, PS1M146V, and TauP301L mice	
		AD patients	N/A	
Microglia and macrophages	Ketone bodies usage: acetoacetate (AcAc), 3-β-hydroxybutyrate (BHB)	AD patients	N/A	[110-112]
		Mouse	Primary mesencephalic neuron-glia cultures	
Microglia-neuron cultures	Alterations in glutamate metabolism	Human	Clinical diagnosis of cognitive status at time of death	[156]
Astrocytes	Mitochondrial complex III-derived reactive oxygen species activation	AD mouse model	5xFAD mice	[115]
		Mouse (B6/SJLF1J genetic background)		
Astrocytes	Glutamine oxidation and impairment in glutamine synthesis	AD mouse models	APPswe/PSEN1dE9 mice	[113, 114]
			5xFAD mice	
Astrocytes	Dysfunctional cholesterol metabolism	AD mouse models	APPswe/PSEN1dE9 mouse	[30, 67]
			5xFAD mouse	
Macrophages	Glycolysis	Various mouse models	C57BL/6J mice	[99-101]
			APP/PS1 APP/PS1/NLRP3^-/-^, Caspase-1^-/-^, APP/PS1/Caspase-1^-/-^	

## 6. Parkinson’s disease

About 1% of people over the age of 60 years suffer from Parkinson's disease (PD) [116]. PD is a progressive neurodegenerative disease that, over time, results in the loss of dopaminergic neurons, as well as an abnormal accumulation of synuclein proteins in the remaining neurons [117]. Typically, motor symptoms do not appear until 70-80% of the dopaminergic neurons in the nigrostriatal dopamine tract of the brain have already been destroyed [116]. PD represents the interaction between genotype, lifestyle, diet, mitochondrial health, drug therapy, and environmental factors [92, 118]. Thus, a metabolome analysis could provide insight into the molecular mechanisms underlying PD and identify potentially predictive biomarkers [119-121]. Interestingly, metabolic dysregulations observed in the serum can distinguish newly diagnosed PD patients from controls with high accuracy. Moreover, plasma metabolome changes have been linked to the progression of PD [122, 123]. However, whether metabolomics can predict PD remains unclear.

**Figure 4.**
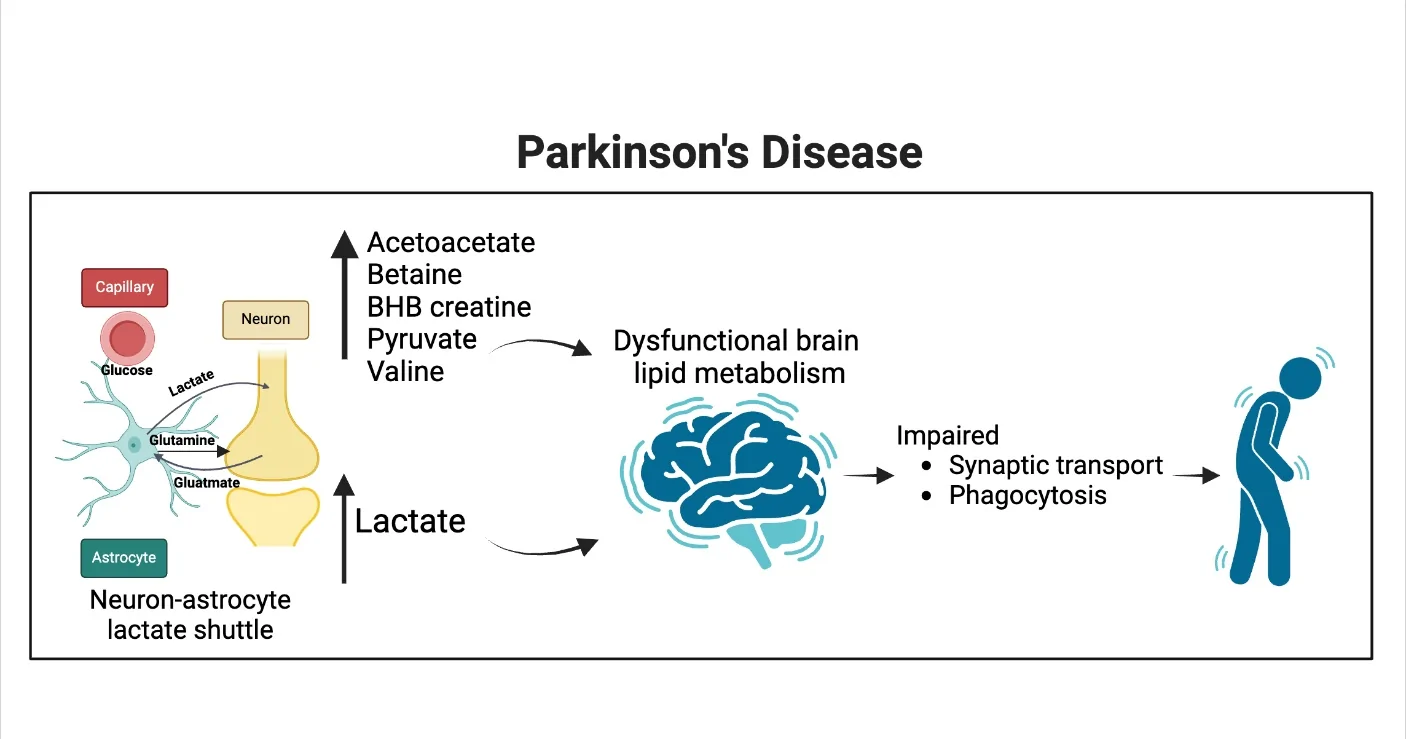
**Immunometabolic dysfunction in Parkinson's disease**. Changes in metabolites such as acetoacetate, betaine, beta-hydroxybutyrate (BHB), creatine, pyruvate, valine, and lactate in the brain play a crucial role in energy metabolism. Impairments in lipid metabolism can disrupt synaptic vesicle transport, endosome-lysosome interactions, and phagocytosis, leading to the formation and stabilization of toxic synuclein oligomers.

Glycolysis and its associated metabolites are increased in PD. One study found that a composite biomarker for PD can be constructed using the profiles of six metabolites: acetoacetate, betaine, BHB, creatine, pyruvate, and valine.[124] Furthermore, lactate levels were increased in the nucleus accumbens (NAcc) and dorsal striatum (DS), which could potentially indicate an increased activity of the astrocyte-neuron lactate shuttle [125, 126]. The contribution of these metabolites to PD pathology is summarized in Figure 4. Researchers have also identified several high-risk metabolism genes in PD patients, including those associated with mitochondrial decline and aggregation of proteins and lipids [127]. Besides the well-known roles lipids play in membrane structure, many also serve as signaling molecules. These lipids are critical regulators of membrane function and fluidity, which help potentiate cellular processes like synaptic vesicle transport, endosome-lysosome interactions, and phagocytosis. Furthermore, reduced lysosomal lipid substrate catabolism disrupts functions critical to clearing neurotoxic proteins like α-synuclein This disruption is further complicated by the binding of α-synuclein to lipid membranes, which can alter their structure and function. In addition, the binding of synuclein monomers to lipids can result in toxic synuclein oligomers that further exacerbate disease pathology [128].

Impairments in lipid metabolizing enzymes may also directly contribute to PD pathology. A meta-analysis of targeted lipid studies suggested that higher levels of total serum triacylglycerol and cholesterol reduce the risk of PD [110]. Likewise, some of the top pathways dysregulated in PD that can be measured in the serum include sphingolipid and glycerophospholipid metabolism, insulin resistance, and OXPHOS [129]. Further characterization of the PD lipidome is required to understand how PD is associated with lipids and their metabolic pathways.

## 7. CNS injury

**Figure 5.**
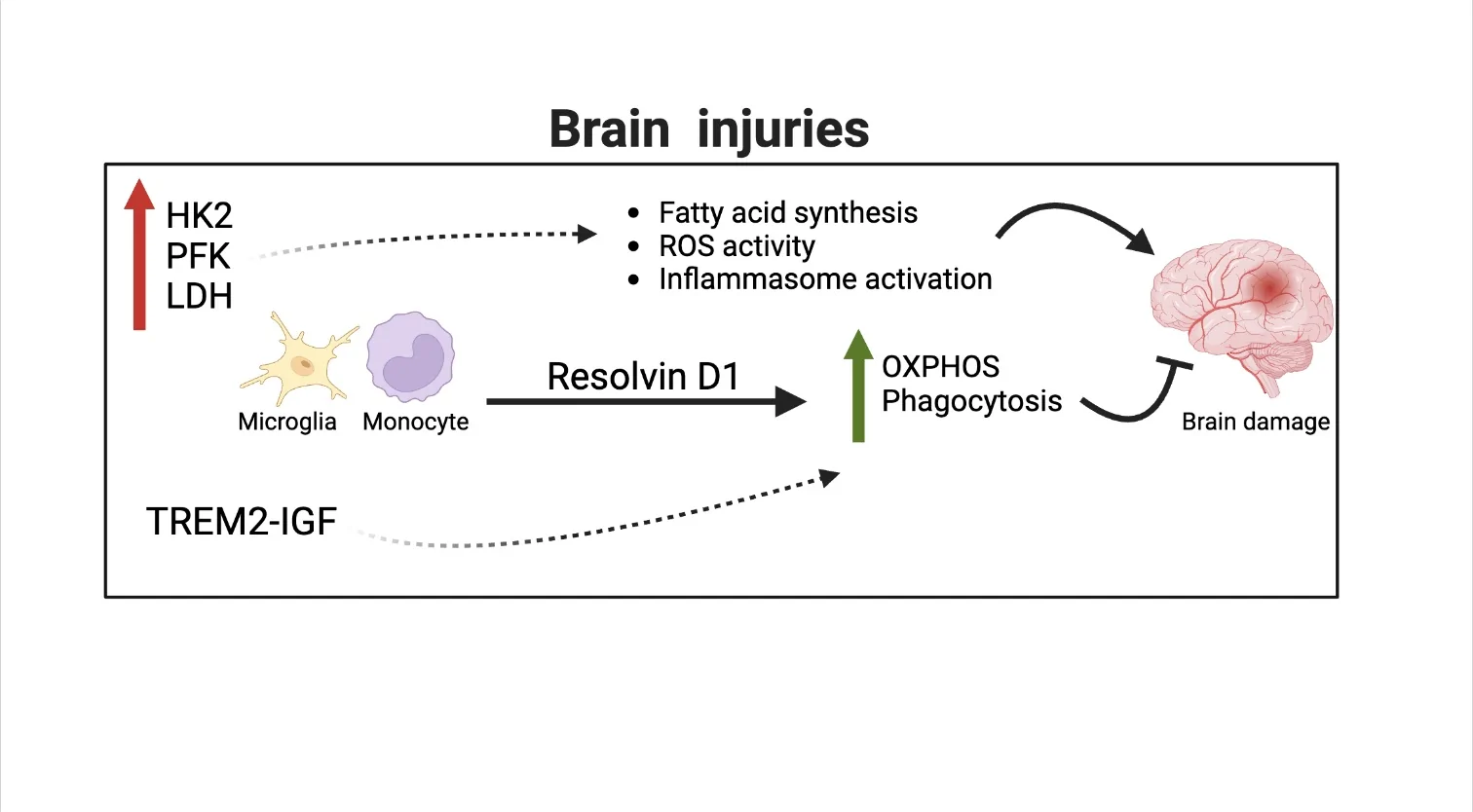
**Immunometabolism dysfunction in CNS injury**. The mechanisms shown here highlight the impact of trauma or damage to the brain, such as in traumatic brain injury (TBI), spinal cord injury (SCI) or ischemic stroke. Collectively, studies have shown an increase in hexokinase (HK2), phosphofructokinase (PFK), and lactate dehydrogenase (LDH) activities after injury, indicating altered glucose metabolism. These pathologies also cause increased fatty acid synthesis, elevated reactive oxygen species (ROS) activity, and inflammasome activation. Resolvin D1 shifts microglial metabolism from glycolysis to oxidative phosphorylation (OXPHOS), enhancing ATP production. High expression of TREM2 and IGF1, along with enhanced oxidative phosphorylation and phagocytic activity. Mechanistically, resolvin D1 (RvD1) and TREM2-IGF1 pathways have been shown to enhance neuroprotection in animal models of brain injury.

CNS injury includes traumatic brain injury (TBI), spinal cord injury (SCI), and stroke. Collectively, the impact of immunometabolic changes on disease pathology in these models is less studied than neurodegenerative disorders; however, *in vivo* evidence strongly suggests that the infiltration of systemic macrophages and endogenous microglia may play a key role in removing debris after these CNS injuries [130-132]. Numerous studies have shown that metabolic reprogramming is a hallmark of macrophage function and phenotype. Injuries to the CNS activate resident microglia and recruit peripheral monocytes. Blood-derived monocytes and activated microglia collectively contribute to progressive neurodegeneration, repair, and protection [133]. In CNS injury, proinflammatory macrophages migrate to the site of injury and, upon arrival, generate ROS to degrade bacteria, synthesize prostaglandins and cytokines utilized in phagocytosis, and have a high membrane turnover [53, 74]. Each of the macrophage functions has distinct metabolic requirements, such as quick energy utilization (glycolysis), NADPH production to increase ROS activity, and fatty acid synthesis [134]. Anti-inflammatory macrophages primarily utilize OXPHOS as their energy source and require sustained activation to synthesize growth factors and transcribe tissue repair genes. On the other hand, proinflammatory macrophages have a higher propensity toward mitochondrial dysfunction and have very urgent energy requirements, so they tend to more often utilize glycolysis as their preferred energy source [67, 135]. Additionally, microglia increase their expression of glycolytic enzymes in response to pro-inflammatory stimuli, including hexokinase 2 (HK2), phosphofructokinases (PFK), and lactate dehydrogenases (LDH) [136, 137]. Figure 5 summarizes the putative signaling pathways that link these enzymes to immune cell activation and subsequent brain injury.

TBI, SCI, and ischemic stroke exhibit some shared pathophysiological mechanisms related to immunometabolism. Macrophages and microglia produce high levels of ROS and pro-inflammatory cytokines. As part of innate immunity, macrophage-derived ROS function acts as an antimicrobial defense; however, in CNS environments, ROS production indicates a pro-inflammatory phenotype that permanently damages neuronal structures. The generation of ROS after ischemic stroke can further activate both CNS inflammatory reactions and NLRP3 inflammasomes, leading to neuronal cell damage, dysfunction, and brain edema.[138] Mitochondrial dysfunction also plays a significant role in the activation of NLRP3 inflammasomes and microglia after stroke. Re-establishing blood flow to a previously occluded area causes additional damage since it allows for further infiltration of neutrophils, a dysregulated microenvironment, and the buildup of ROS, which leads to the damaging inflammatory effects mentioned previously. In rats, medications that protected the mitochondria from oxidative stress or inhibited the over-activation of the NLRP3 inflammasomes following ischemic stroke were shown to help prevent further damage [139]. During inflammation, macrophages and microglia redirect glycolytic intermediates to the oxidative phase of the pentose phosphate pathway (PPP) using NADPH oxidase (NOX) to generate ROS. Studies have shown that increasing NOX activity after TBI and SCI leads to increased ROS production by macrophages/microglia. Thus, it has been hypothesized that inhibiting NOX could improve outcomes after TBI or SCI [140-142].

The effects of permanent middle cerebral artery occlusion (pMCAO) were assessed in mice administered chemokine receptor 1 (CXCL1), also known as fractalkine. Neurons express CXCL1 to signal microglia as well as macrophage migration and infiltration. CXCL1 polarizes microglia from a pro- to anti-inflammatory phenotype *in vivo*, in tandem with reducing ischemic damage. CXCL1 treatment decreased the expression of several glycolysis-related genes and increased the expression of OXPHOS-associated genes, including PPAR-γ coactivator-1 beta (PGC1β) and Solute Carrier Family 25 Member 15 (SLC25A15) [143, 144]. Studies investigating HK2 in experimental stroke have provided direct *in vivo* evidence for glycolysis driving pathological microglia activation and inflammation. In contrast, blocking HK2 activation after transient MCAO reduced infarct area, decreased microglial activation, and decreased the release of the proinflammatory cytokine, IL-1β [103].

More recent studies have emphasized the role of microglia metabolism in brain injury by investigating the role of resolvin D1 (RvD1). RvD1 is a lipid mediator derived from docosahexaenoic acid (DHA), promotes microglial phagocytosis of neutrophils in ischemic stroke by reprogramming microglial energy metabolism. RvD1 shifts microglial metabolism from glycolysis to oxidative phosphorylation (OXPHOS), thereby enhancing ATP production. This energy boost facilitates the clearance of neutrophils and reduces neuroinflammation after ischemic stroke. Additionally, RvD1 stimulates glutaminolysis, which supports OXPHOS in an AMPK-dependent manner, contributing to improved microglial function and neuroprotection [145]. Another microglial regulator in ischemic stroke is the TREM-IGF1 signaling axis. In a model of pMCAO, investigators identified a subcluster of microglia in ischemic brain tissue that exhibited high expression of TREM2 and IGF1, along with enhanced oxidative phosphorylation and phagocytic activity. Depletion of microglia exacerbated ischemic brain damage, while repopulation of microglia with elevated TREM2 and IGF1 levels conferred neuroprotective effects. Mechanistically, these studies support the concept that IGF1 acts as a downstream effector of TREM2, and that boosting microglial IGF1 expression enhanced neuroprotection by increasing glucometabolic function [146].

## 8. Conclusions and future directions

This review provided an overview of how shifts in metabolism, changes in metabolic substrates, and accumulation of metabolic intermediates can impact immune cells in brain disorders associated with aging and neurodegenerative disease. Classically, studies of immunity and inflammation have been centered on how the host responds to pathogens, whereas the study of metabolism was focused on the regulation of energy production for cellular function in an organism. In recent years, connecting these two concepts has been at the forefront of understanding and potentially treating a wide variety of neurodegenerative diseases or CNS injuries. Researchers have now extended their initial *in vitro* findings to *in vivo* studies which have provided more conclusive evidence that immunometabolic disturbances contribute to the mechanisms of neurodegeneration and brain injury. Nonetheless, more research is needed to uncover the mechanisms underlying metabolic reprogramming of immune cell responses in the CNS and to determine whether immunometabolic targets can be used therapeutically in CNS injuries and diseases. One important area is understanding the contributions of other cells in the neurovascular unit such as brain endothelial cells, pericytes, and neurons to brain immunometabolism in human health and disease. Additional outstanding basic and translational research questions that must be addressed to fill critical research gaps are:

- How do resident and infiltrating brain immune cells utilize metabolism to elicit unique effects when metabolic pathways are largely conserved across all cells?
- What are specific sex-specific responses in brain immunometabolism and how will these differences influence the development of immunometabolism-based therapeutics?
- What insights can genetically modified mice mouse provide about manipulating metabolic pathways in immune cells to understand neuroinflammation and neurodegeneration?
- Can longitudinal metabolic profiling of peripheral immune cells reveal novel insights on neurodegenerative disease progression?
- How can multi-omics approaches, i.e. transcriptomics, proteomics, and metabolomics, be integrated in animal models and patients to better understand brain immunometabolism?

These questions highlight potential avenues for enhancing the understanding and treatment of neurodegenerative diseases through immunometabolic regulation. We anticipate that new and exciting discoveries are imminent in treating neurological diseases by targeting metabolic enzymes and metabolite intermediates to modulate immune responses.
